# Anesthetic management of patients with difficult intubation due to temporomandibular joint osteoarthritis: A case report

**DOI:** 10.1097/MD.0000000000036956

**Published:** 2024-01-12

**Authors:** Changle Rao, Guihua Huang, Fangfang Mu, Zhengquan Tan, Jie Yuan

**Affiliations:** a Department of Anesthesiology, The Third Affiliated Hospital of Zunyi Medical University, Zunyi, China; b Department of Anesthesiology, Affiliated Hospital of Zunyi Medical University, Zunyi, China; c Department of Pain Medicine, Affiliated Hospital of Zunyi Medical University, Zunyi, China.

**Keywords:** difficult airway, lightwand, temporomandibular joint osteoarthritis

## Abstract

**Introduction:**

Temporomandibular joint osteoarthritis (TMJOA) affects 8% to 16% of the global population, yet TMJOA remains relatively underappreciated clinically. To anesthesiologists, who is concerned about patient safety, adequate preoperative evaluation and preparation, as well as individualized anesthetic management of patients, are necessary. Therefore, the anesthesiologist should be alert for difficult airways due to TMJOA, have a full and comprehensive understanding of the disease, and possess the appropriate expertise for difficult airway intubation.

**Case presentation:**

A 52-year-old female patient was scheduled for laparoscopic operation of uterine adnexa under general anesthesia. The patient preoperative evaluation showed only 1 finger width of mouth opening, and the computed tomography scan showed bilateral temporomandibular arthritis, which was evident on the right side. Intraoperatively, the expected airway difficulties occurred, and the anesthesiologist opted to use lightwand intubation, which was ultimately successful in 1 pass without any complications.

**Conclusion:**

Intubation using a lightwand for patients with difficult intubation due to TMJOA is a very effective intubation modality.

## 1. Introduction

Temporomandibular joint osteoarthritis (TMJOA) is a degenerative disease characterized by progressive cartilage degeneration, subchondral bone destruction, bone redundancy formation, and synovitis.^[1]^ The main clinical symptoms of TMJOA are joint pain, dysfunction, and limited opening, which can severely diminish the patient quality of life.^[2]^ When TMJOA is severe, restricted opening may lead to difficult intubation, posing a major airway management challenge for the anesthesiologist and requiring a detailed pre-anesthetic assessment, especially regarding the degree of opening. Here, we report the case of a 52-year-old female patient with severe mouth opening restriction due to TMJOA who was successfully intubated using a lightwand.

## 2. Case presentation

**Figure 1.**
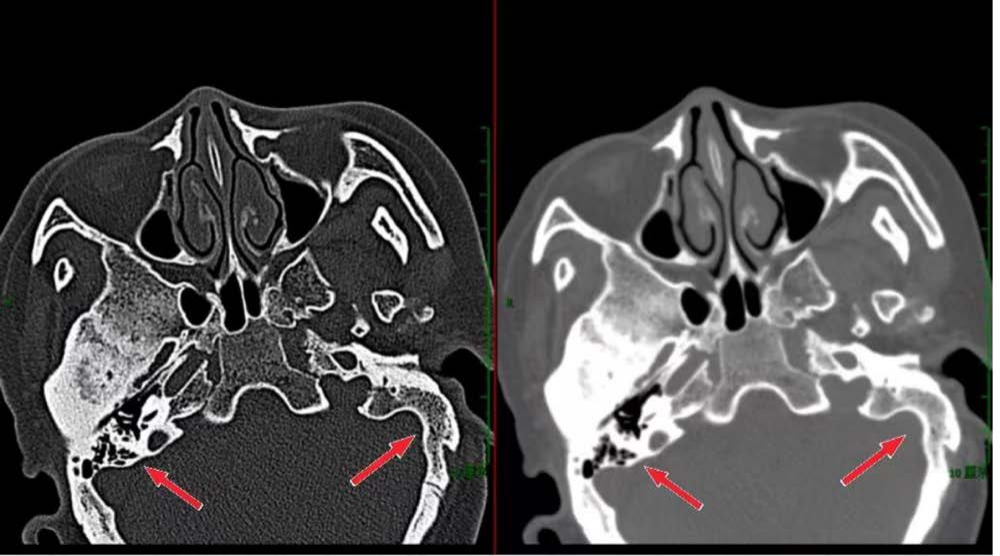
Mandibular bone/section (spiral) (CT imaging + 3D reconstruction): Temporomandibular joint osteoarthritis on both sides, with significant symptoms on the right side. CT = computed tomography.

A 52-year-old female, 153 cm tall, weighing 57 kg, American Society of Anesthesiologists score of 2, was scheduled to undergo lumpectomy for adnexal uterine surgery. The preoperative laboratory findings showed that her complete blood count was normal with hemoglobin at 138 g/L, erythrocyte volume at 42.4%, WBC count at 3.8 × 10^9^/L, and platelet count at 134 × 10^9^/L. There were no significant abnormalities in coagulation, renal, and liver functions. The electrocardiogram (ECG) showed a normal range ECG. The computed tomography (CT) examination of the mandible/part (spiral) (CT imaging + 3D reconstruction) and neck (spiral) (CT plain + 3D reconstruction) showed that the patient had arthritis of the temporomandibular joint, which was obvious on the right side, and degenerative degeneration of the cervical spine with central type herniation of cervical 3 to 5 disc (Fig. 1).

**Figure 2.**
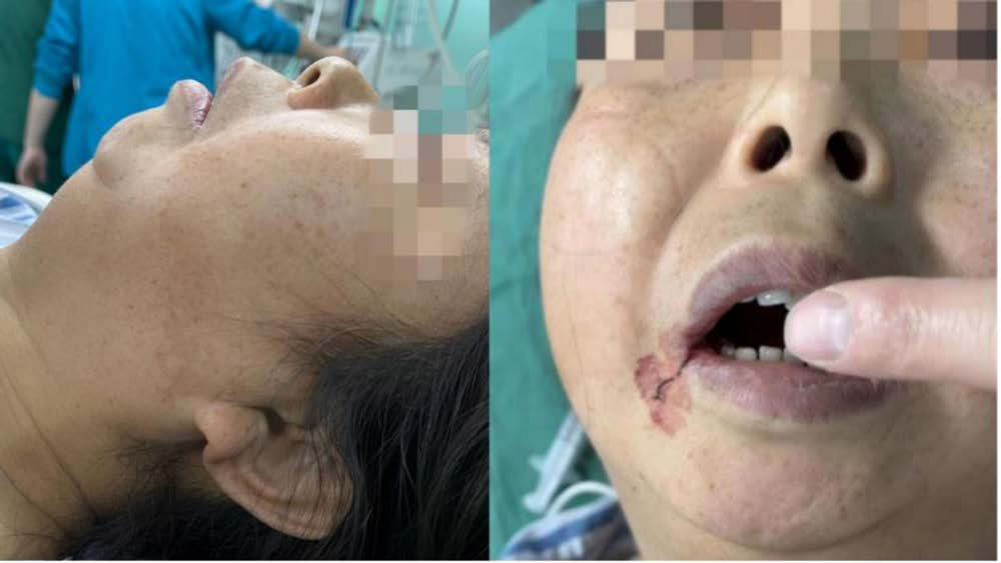
Open mouth examination (maximum mouth opening, distance between upper and lower incisors is only 1 finger).

During the preoperative visit, the patient had a Mallampati grade IV due to severe mouth opening restriction with only 1 finger of mouth opening (Fig. 2). After a thorough preoperative evaluation and preparation, the patient was prepared for surgery and anesthesia after discussing the associated risks with the surgeon, the patient, and her family. Vital signs before induction of anesthesia included a heart rate of 82 beats per minute, arterial blood pressure of 120/80 mmHg, and oxygen saturation of 99% on room air. After preoxygenation with 100% oxygen, anesthesia was induced with 20 mg of etomidate, 2 mg of midazolam, 25 µg sufentanil, and 12 mg cisatracurium benzoate. After induction, laryngoscopy was first attempted with a visual laryngoscope, but the attempt failed because the mouth opening was too small to place the visual laryngoscope lens in. Then, the anesthesiologist tried to guide the intubation via the lightwand. The left hand lifted the lower jaw, and the right hand inserted the lightwand along the pharynx. The tip of the lightwand was bent 90 degrees along the tongue root at the midline, and the red light at the tip of the lightwand was transmitted and positioned at the cricothyroid membrane of the neck. The tracheal tube was then gently inserted into the neck where there was light, and finally, the tube was successfully intubated. Heart rate, oxygen saturation, ECG, end-tidal carbon dioxide, invasive arterial blood pressure, and temperature were monitored intraoperatively. Additionally, intraoperative anesthesia was maintained with a combination of remifentanil 0.1 ug·kg^−1^·min^−1^, dexmedetomidine 0.5 µg·kg^−1^·h^−1^ continuous pumping, and 2% sevoflurane by static inhalation. The operation progressed smoothly with a fluid intake of 1000 mL of sodium lactate Ringer solution and blood loss of 50 mL. The operation lasted 1.25 hours. After extubation, the patient was transferred to the postanesthesia care unit. After awakening, the patient was in good condition and returned to the ward without any significant perioperative complications.

## 3. Discussion

TMJOA is a common condition that affects 8% to 60% of the population (the exact prevalence of the disease is difficult to determine since many asymptomatic patients exhibit imaging manifestations of TMJOA) and is more common in women.^[3–5]^ The main clinical symptoms of TMJOA are joint pain, functional impairment, and restricted opening.^[2]^ Although these symptoms suggest TMJOA, the diagnosis depends on imaging tests, which reveal cortical bone erosion, flattening of the joint compartment, sclerosis, bone formation, and subcortical cyst formation.^[6]^

It is important to note that a direct relationship between radiological findings and TMJOA symptoms may not always be present.^[7]^ In fact, many patients with TMJOA are asymptomatic.^[8]^ The etiology of most TMJOA cases is complex, multifactorial, or unknown. Overloading of the temporomandibular joint is currently believed to be one of the major causes of the disease, including severe malocclusion, bony jaw asymmetry, and muscle overuse. However, it is difficult to attribute most cases of TMJOA to overloading, and the pathogenesis of TMJOA remains controversial.^[9]^ TMJOA leads to reduced quality of life for patients, and since articular cartilage has limited ability to repair itself, it is one of the most difficult joint diseases to cure, with no treatment available for complete remission.^[6]^ In the case of the patient described above, the combination of clinical symptoms and imaging findings was consistent with TMJOA. Inquiring about the patient past medical history revealed that she had fallen and injured her jaw when he was 2 years old. This injury may have led to osteoarthritis of the temporomandibular joint due to trauma, which can cause osteoarthritis of the bone and jaw. The anesthetic evaluation focused on the degree of difficult airway and altered mouth opening. During intubation, if the mouth opening is too small, it can lead to difficult laryngoscopic exposure. This patient had a significantly restricted mouth opening (only 1 horizontal finger width) and Mallampati grade IV, both of which suggest that tracheal intubation will be difficult.^[10]^ During the preoperative evaluation, the laryngeal lens cannot be placed if the mouth opening is <3 cm or 2 fingers across. This can lead to difficult laryngoscopic visualization, suggesting difficult airway management. The Mallampati grade is the most common method of airway assessment. A Mallampati grade III (only soft palate visible) or a Mallampati grade IV (only hard palate visible) is an independent threat factor for difficult mask ventilation, and Mallampati grade III to IV suggests a difficult airway.^[11]^ Therefore, the pathophysiological changes brought about by TMJOA pose a huge challenge for anesthetic and airway management. While intubation used to be performed under plain laryngoscopy in most cases, today there is an emphasis on visualized non-blind intubation techniques. This is driven by the development of new imaging techniques in difficult airway management, which gives visual laryngoscopy a huge advantage in clinical applications.^[12]^ However, the use of video laryngoscopy may be limited in patients with restricted opening and cervical ankylosis.^[13]^ Tracheal intubation using a lightwand is a good alternative for patients with restricted opening and cervical ankylosis. Therefore, in this case, we chose to use a lightwand-guided tracheal intubation that is easy to perform and does not require exposure of the vocal cords. The intubation was successful in a single pass, and intraoperative ventilation was good. The lightwand-guided intubation technique is widely used for difficult airway intubation because of its low impact on patient vital signs and low patient complication rate. One study reported that 96.8% to 100% of patients with difficult airways were successfully intubated when using lightwand intubation. It is recommended that when performing lightwand intubation, the anesthesiologist should pay attention to the optimal point of transmitted light in the patient neck, which should be located approximately 1 cm below the superior border of the cricoid cartilage.^[14]^ Secondly, tracheal tube removal is a dangerous phase of anesthesia, and patients with difficult airways should be extubated with adequate timing and gentle movements to prevent complications of tracheal tube removal and avoid leading to reintubation.

As an anesthesiologist, one should be alert to difficult airways due to osteoarthritis of the temporomandibular joint. Successful anesthetic management in cases of TMJOA requires a thorough understanding of anatomical variations as well as expertise in difficult airway intubation. The presence of a difficult airway poses a significant challenge to perioperative airway management, and improper management of a difficult airway can lead to severe brain injury and death; therefore, anesthetic management of patients with difficult airways must be of concern to anesthesiologists to improve the success rate of the first attempt at tracheal intubation, to improve patient safety during airway management, and to minimize or avoid adverse events.^[15,16]^ It is also recommended that the patient or responsible physician be informed of any airway difficulties encountered and that the presence and condition of airway difficulties be documented in the medical record.

Typically, as recommended by the American Society of Anesthesiologists, special attention should be paid to the degree of opening, Mallampati classification, Thyromental distance, or other methods of assessment when performing a pre-anesthetic evaluation. In conclusion, a careful preoperative examination of each patient is essential for safe anesthetic management, and an appropriate and careful assessment of the airway can predict and avoid difficult intubations.

## 4. Concvlusion

Intubation using a lightwand for patients with difficult intubation due to TMJOA is a very effective intubation modality.

## Acknowledgments

The authors sincerely thank the family for giving permission to report this case.

## Author contributions

**Data curation:** Zhengquan Tan, Changle Rao, Fangfang Mu.

**Resources:** Guihua Huang.

**Writing – original draft:** Changle Rao, Jie Yuan.

**Writing – review & editing:** Guihua Huang, Fangfang Mu, Zhengquan Tan, Jie Yuan.
